# Systemic Capillary Leak Syndrome: Is Methylene Blue the Silver Bullet?

**DOI:** 10.1155/2014/141670

**Published:** 2014-12-07

**Authors:** Michele Umbrello, Marco Gardinali, Davide Ottolina, Giancarlo Zanforlin, Gaetano Iapichino

**Affiliations:** ^1^Unità Operativa di Anestesia e Rianimazione, Azienda Ospedaliera San Paolo, Polo Universitario, Via A. Di Rudinì 8, 20142 Milano, Italy; ^2^Unità Operativa di Medicina IV, Azienda Ospedaliera San Paolo, Polo Universitario, Via A. Di Rudinì 8, 20142 Milano, Italy; ^3^Dipartimento di Fisiopatologia Medico-Chirurgica e dei Trapianti, Università degli Studi di Milano, Via F. Sforza 35, 20122 Milano, Italy

## Abstract

*Background*. Systemic capillary leak syndrome (SCLS) is a rare disorder characterized by unexplained, recurrent episodes of transient, abrupt increase in endothelial permeability, leading to severe hypotension, generalized edema, and hemoconcentration. *Case Report*. We report the case of a patient suffering from systemic capillary leak syndrome and present a possible interpretation of the pathophysiology of this condition. Besides the classical triad of hypotension, edema, and hemoconcentration, we recorded increased levels of methemoglobin, an index of NO overproduction. We present a possible interpretation of the pathophysiology of this condition based on the fast and complete reversal of symptoms after methylene blue administration (which opposes NO-induced effects) and speculate that increased NO levels could be implicated in the pathophysiology of the capillary leak phase. *Why should an emergency physician be aware of this?* The safety of this treatment and its fluid- and cathecolamine-sparing effect deserve consideration and further research.

## 1. Introduction

Systemic capillary leak syndrome (SCLS) is a rare disorder characterized by unexplained, recurrent episodes of transient, abrupt increase in endothelial permeability, leading to severe hypotension, generalized edema, and hemoconcentration [1]. Both etiology and pathogenesis are currently unknown, and systematic research is clearly limited by the rarity of the disease. Several hypotheses have been formulated, but clear evidence is lacking to support any.

We describe the improvement of one patient with SCLS in two occasions with acute IV administration of methylene blue after failure of the medications commonly used in this setting. We present a possible interpretation of the pathophysiology of this response to therapy.

## 2. Case Presentation

In March 2013, a 56-year-old man, otherwise healthy, suffered from a cold and low-grade fever and gradually developed oliguria and weight gain. The general practitioner prescribed some biochemical tests, which resulted normal except for serum albumin 3 g/dL and a monoclonal IgG/*κ* peak. An empirical course of broad-spectrum antibiotic and furosemide was started.

Ten days later, he developed massive peripheral edema, bilateral pleural and pericardial effusion, and ascites. Body temperature was normal, arterial blood pressure (ABP) was 90/60, and heart rate was (HR) 120/min. Blood tests showed albumin 2.3 g/dL, creatinine 1.4 mg/dL, sodium 129 mEq/L, hemoglobin (Hb) 22 g/dL, and hematocrit (Ht) 62%. Crystalloid loading was started, but despite a positive balance of about 7000 mL over 12 hours, ABP kept falling down to 70/40 and the patient became anuric. Norepinephrine (NE) up to 0.2 mcg/kg/min was then added, with ABP only rising to 80/60. Despite the presence of only tachycardia as a SIRS sign (WBC 11780/mm^3^, PaCO2 37 with RR 18/min, core body temperature 36.4°C, HR 120/min), the case was interpreted as refractory septic shock of unknown origin (cultures later resulted negative), and empirical treatment with methylene blue (1.5 mg/kg IV bolus over 1 hour) was given. Soon after the end of the infusion, ABP rose to 140/80, HR 75/min, diuresis restarted, and norepinephrine was suspended in 2 hours. Renal function improved and edema and effusions reduced. The patient was discharged home with no therapy, whilst awaiting more tests.

In the following months he experienced two less severe episodes, requiring admittance to a nephrology ward for fatigue, hypotension, peripheral swelling, oliguria, hypoalbuminemia, and hemoconcentration. During the course of these admissions, a series of tests were run, showing normal levels of complement and C1-esterase inhibitor, negative cancer markers, no autoantibodies; immunoglobulin levels, including IgE, were in the normal range. Absence of diarrhea excluded a protein losing enteropathy. Renal biopsy yielded no alterations, and brain MR excluded pituitary lesions. Thyroid and adrenal functions were normal. Bone marrow biopsy excluded malignancies. Subcutaneous fatty tissue biopsy excluded systemic amyloidosis. POEMS syndrome was excluded by the absence of any sign of polyneuropathy. Determination of vascular endothelial growth factor (VEGF) showed increased levels (1429 pg/mL—normal values <450 pg/mL). The recurrence of episodes of hypotension, hypoalbuminemia, and hemoconcentration and the presence of a monoclonal IgG/*κ* peak led to the diagnosis of systemic capillary leak syndrome: prophylactic treatment with theophylline 300 mg bid was started.

**Figure 1:**
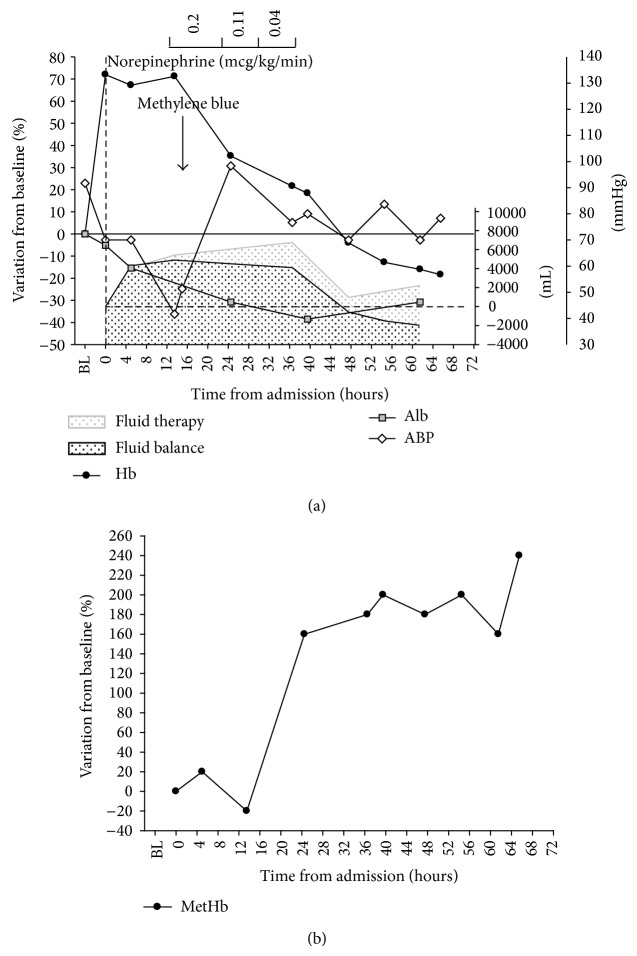
(a) Time course of blood pressure, haemoglobin, and albumin levels during the acute episode and effects of therapeutic interventions. (b) Time course of methemoglobin during the acute episode. BL: baseline (3 months before the acute episode), Hb: haemoglobin (% variation form baseline), Alb: albumin (% variation form baseline), ABP: mean arterial blood pressure (absolute value), and MetHb: methaemoglobin (% variation form baseline).

In October 2013, the patient again experienced symptoms of a cold similar to those before the first admission. On arrival to the hospital, he was hypotensive and tachycardic; blood tests (Table 1) showed severe hemoconcentration with normal albumin levels. He was oliguric and had peripheral edema. Again, SIRS was not present. Echocardiography showed a pericardial effusion 18 mm, with no sign of tamponade, normal valves, hypertrophic left ventricle with slightly reduced end-diastolic volume (70 mL), and 60% ejection fraction, normal diastolic pattern, TAPSE 20 mm. Inferior vena cava diameter was unmeasurable. Fluid loading with crystalloids was started, but despite a positive balance of 5500 mL/12 h, ABP kept falling (Figure 1) and urine output remained low. NE was started and rapidly increased up to 0.2 mcg/kg/min, with minimal response in terms of ABP. ABG showed increasing levels of lactic acid and methaemoglobin (Table 1). Organ failure was quantified, yielding a SOFA score of 8, only depending, however, from complete failure of cardiocirculatory and renal system (norepinephrine 0.2 mcg/kg/min, anuria) with no sign of compromise of any other organ (GCS 15, bilirubin 1.1, platelet count 180000, PaO2 81 mmHg at room air).

Given the lack of response to treatment, bearing in mind the previous anecdotal response, methylene blue was administered (1.5 mg/kg IV bolus over 1 hour), and ABP again rapidly rose to 135/90, allowing for NE termination 8 hours later. Urine output gradually increased, allowing for the negativization of fluid balance and concurrent recovery of renal function. Haemoglobin levels normalized over a 48-hour period, while albumin remained low (Figure 1). The patient was thereafter discharged home.

## 3. Discussion

We describe a case of SCLS that responded twice—one time as a rescue for a suspected diagnosis of septic shock, while the second time intentionally to the administration of methylene blue. In both episodes, the patient had hypotension (with ongoing further reduction during the first 12 hours) and signs of severe haemoconcentration (Hb and Ht about 70% higher than his own baseline normal values) and needed significant amount of NE, despite positive fluid balance of >5 liters in 12 h, in line with the well-known poor response to fluid and vasoactive drugs of SCLS [1]. On the contrary, albumin concentration was not increased, and it further decreased during the first hours, suggesting a shift towards the extracellular space due to increased endothelial permeability. Accordingly, transcapillary escape rate of radio-labeled albumin was found to be elevated [2] in similar cases. Organ perfusion was inadequate, as highlighted by the reduced urinary output and the increase of plasma lactic acid; however, the absence of a profound metabolic acidosis and the only presence of tachycardia as a sign of SIRS could have raised doubts on the diagnosis of refractory septic shock.

Actual management of the acute shock phase in this condition is based on support of vital functions with IV fluids, including colloids and vasopressors [3]. We avoided albumin administration as extravasation may lead to increased interstitial colloid osmotic pressure, with further reduction of circulating blood volume, and to reduced de novo synthesis [4]. Infusion of pentastarch or dextrans [5] which could seal the negatively charged endothelial fenestrations [4], were unavailable for clinical use in Italy. Echocardiographic assessment showed signs of both hypovolemia (complete collapse of the inferior vena cava) and loss of systemic vascular resistance (hypotension despite preserved systolic function, as indicated by the normal values of ejection fraction and TAPSE). After about 13 hours of standard treatment, we decided to administer methylene blue and consequently observed the rapid reversal of hypotension, a concomitant gradual reduction of Hb and Ht back to baseline levels, an increase in urine output, a negative fluid balance, and resolution of edema. All these signs are compatible with normalization of endothelial permeability. Albumin levels, however, remained low, likely because the escaped molecules only partially flow back into vessels and are degraded in tissues [6], while the turnover rate for de novo synthesis can take up to 20 days to restore normal values [7]. Meanwhile, during the second episode, we observed a significant increase of methaemoglobin levels.

Despite severe hypotension, the patient was awake and cooperative; moreover, hypoxemia was not present ad admission, and the lung CT scan excluded pulmonary edema. Usually, during SCLS attacks, musculature and connective tissue are the principal target sites of the extravasation of plasma, while the lungs, brain, and kidneys seem to be infrequently involved [8]; as such, we think that the measurement vascular permeability, such as the pulmonary vascular permeability index derived by transpulmonary thermodilution [9], is of limited help in this phase. However, it is quite common that pulmonary edema develops during the recovery phase, as an iatrogenic side effect of the huge amount of intravenous fluid often administered. In this phase, we can speculate that the availability of such an index might potentially be of help for the evaluation of the effects of therapy.

This patient, as the majority of those described in series and case reports, presented a monoclonal gammopathy [10] and elevated VEGF levels [2]; many Clarkson's disease cases predominantly have IgG-*κ* or IgA-*κ* monoclonal gammopathy [8]: both multiple myeloma and systemic amyloidosis were excluded via abdominal wall fat pad and bone marrow biopsy. POEMS syndrome (polyneuropathy, organomegaly, endocrinopathy, monoclonal gammopathy, and skin changes), which is almost always associated with IgG-*λ* or IgA-*λ*, was excluded by the absence of any sign of polyneuropathy.

The patient was also in long-term treatment with theophylline [11], a drug that increases intracellular cAMP levels. Any possible pathophysiologic interpretation of the response to methylene blue should take these findings into account. In addition to its mitogenic effect, VEGF was shown to increase endothelial permeability, in a process mediated by a NO-dependent increase in cGMP levels [12], the main modulator of increased vascular permeability. Moreover, IL-2 might contribute to the pathogenesis of SCLS [1], based on the fact that IL-2 therapy can develop a leakage syndrome undistinguishable from SCLS [13]. Indeed, increased IL-2 expression was demonstrated on perivascular cells of symptomatic cases of SCLS [14]. Nitric oxide (NO) was suggested as the mediator of IL-2-induced endothelial permeabilization [15], and inhibition of NO synthesis resulted in reversal of IL-2-induced leakage [16]. Hence, the NO system seems to be a common pathway for both IL-2- and VEGF-mediated endothelial permeabilization. The vascular effects of NO are mainly mediated by the activation of soluble guanylate cyclase (sGC), which leads to the synthesis of cGMP; the latter, in turn, acts on several targets eventually causing smooth muscle relaxation [17] and increased endothelial permeability [18]. cGMP-mediated signalling also contributes to vasopressor hyporesponsiveness [19]. Instead, cAMP (increased by theophylline) counteracts with this pathway, protecting the basal barrier function [18].

The response to methylene blue supports the hypothesis that increased NO levels could be implicated in the pathophysiology of the acute capillary leak phase. Methylene blue (3,7-bis(dimethylamino)-phenothiazin-5-ium chloride) is generally used in the setting of methaemoglobinemia [20] and proved effective in reversing hypotension and restoring the response to vasoactive drugs in many different conditions characterized by increased levels of NO (sepsis, anaphylaxis, severe burns, ischaemia-reperfusion injury and liver failure) [21–25]. Methylene blue opposes NO-induced effects mainly by inhibition of sGC [26–30], ultimately reducing the generation of cGMP. Acute methylene blue administration, unlike drugs that increase intracellular cAMP levels (such as theophylline), aims at decreasing cGMP levels to reduce the severity of supposedly NO-mediated attacks.

Although multiple therapies were administered during the clinical management of the patient, both times we recorded a clear temporal relation between methylene blue administration and reversal of the symptoms, leading us to identify this treatment as the main cause for the change in the clinical course. We did not report direct measures of NO levels in the case we described. Indeed, NO has a very short half-life [31]. However, in septic populations, circulating levels of methaemoglobin proved effective indicators of NO overproduction [32]. Methaemoglobin is generated by the reaction of haemoglobin with NO [33]. In the present case, methaemoglobin levels increased up to 250% their baseline value during the course of the episode. We observed a delay from symptoms appearance and methaemoglobin rise: methaemoglobin generation in the presence of elevated NO may require up to several hours to occur* in vitro* [34]; under* in vivo* conditions, this time may even increase, depending on plasma redox state.

At pharmacologic doses methylene blue is a reducing agent via the NADPH-methemoglobin reductase pathway [20]. Methylene blue, when injected intravenously as an antidote, is reduced to leucomethylene blue, which then reduces the heme group from methemoglobin to hemoglobin. However, when given in higher doses, methylene blue may oxidize the ferrous iron of hemoglobin to ferric iron, thus potentially resulting in methemoglobin production. However, the dose we administered in the present case is well within the doses used in the setting of methaemoglobinemia (1-2 mg/kg, repeated up to twice if symptoms of hypoxia fail to subside) [35], thus reducing the possibility that the elevated methaemoglobin levels we recorded were generated by the methylene blue we administered. Elevated levels of methaemoglobin persisted up to the last blood sample taken in the ICU, likely after the effects of methylene blue ended; however, this is not surprising as the elimination half-life of methaemoglobin has been reported to be as high as 15–20 hours [36].

The main limit of the present investigation is its case-report nature. However, the rarity of this condition makes experimental studies difficult to plan, and the incomplete understanding of the underlying mechanisms does not allow for preclinical modeling. Moreover, the hemodynamic response to methylene blue might have simply faced by chance the natural course of the syndrome, which spontaneously reversed. However, the response we observed was similar in two different occasions, and both times it occurred soon after the administration of the drug.

SCLS is a rare yet potentially severe condition, often difficult to recognize and diagnose upon initial presentation. Current therapies are mainly supportive, and the condition is still associated with a 10-year mortality rate of about 30% [1]. We report our experience with a treatment whose safety [37], along with its fluid- and catecholamine-sparing effect deserve consideration and further research.

## Figures and Tables

**Table 1:** Time course of hemodynamic and laboratory parameters at baseline and during the second acute episode.

Time (hours)	BL	0	+5	+13.5	+24.5^*^	+36.5	+39.5	+47.5	+54.5	+61.5	+65.5
ABP (mmHg)	125/75	80/50	90/60	65/30	135/80	110/60	100/70	110/50	129/61	110/50	115/60
HR (1/min)	73	140	134	120	105	100	97	112	98	102	101
CVP (mmHg)	—	—	—	2/−3 (−1)	—	2/−3 (−1)	—	16/7 (12)	—	7/0 (4)	8/4 (6)
Hb (g/dL)	12.5	22.0	22.4	22.7	17.5	15.2	13.4	12	10.9	10.5	10.2
Ht (%)	37.1	67.1	68.2	69	53.4	46.5	41.2	36.9	33.5	32.3	31.5
Albumin (g/dL)	3.9	3.7	3.3	—	2.7	—	2.4	—	—	—	—
Creatinine (mg/dL)	1	1.2	0.9	1.2	1.5		1.2				
pH	7.4	7.4	7.4	7.4	7.35	7.4	7.46	7.46	7.46	7.47	7.43
BE (mmol/L)	1.1	−2.8	−0.8	−4	−0.9	4.9	8.2	9.8	11.4	12.1	10.1
HCO_3_ (mmol/L)	26.4	22.9	24.2	22.6	24	29.6	32.4	34.3	36.1	36.6	35
Lactate (mmol/L)	0.6	1.6	1.7	2.2	2	1.3	1.6	1.2	1	1.1	1.3
Na (mEq/L)	141	134	134	131	137	139	141	142	143	143	143
K (mEq/L)	3.7	4.3	4.6	5.6	4.2	3.7	3.8	3.4	3.3	3.5	4.1
MetHb (%)	—	0.5	0.6	0.4	1.3	1.4	1.5	1.4	1.5	1.3	1.7

BL: baseline (3 months before the acute episode). First line refers to hours after admission. ^*^Denotes methylene blue administration.

ABP: arterial blood pressure; HR: heart rate; CVP: central venous pressure; Hb: haemoglobin; Ht: hematocrit; MetHb: methaemoglobin; BE: base excess; HCO_3_: bicarbonate; Na: sodium; K: potassium.

## Abbreviation List

ABP: Arterial blood pressure
cAMP: Cyclic adenosine monophosphate
cGMP: Cyclic guanosine monophosphate
Hb: Hemoglobin
HR: Heart rate
Ht: Hematocrit
NE: Norepinephrine
NO: Nitric oxide
SCLS: Systemic capillary leak syndrome
sGC: Soluble guanilate cyclase
VEGF: Vascular endothelial growth factor.
